# The Period of Puberty and the Teeth

**Published:** 1887-01

**Authors:** Daniel T. Nelson


ARTICLE III.

### THE PERIOD OF PUBERTY AND THE TEETH.

BY DR. DANIEL T. NELSON.

[Retiring President of the Chicago Gynæcological Society, in his address, speaks as follows about the Decay of Teeth at Puberty, Proper Developments of Sexual Organs, Mothers' Food, Tight Lacing, &c.]

There is, I know, a law of compensation, whereby if one organ or system of organs is disabled or over worked, another will try to aid it to do its work, as the skin and alimentary canal when the kidneys are disabled, or the fingers and the ears when the eyes are useless; but these are conditions of disease, not health; of pathology, not physiology.

Tried by this law, then, what are the conditions necessary for the normal development of the reproductive organs? Plainly, the normal development and activity of all the other organs and system of organs, and conversely, the other systems of organs can only attain their highest functional activity when the sexual organs are perfectly developed. During the period of puberty, then, from 10 to 18 years, or more commonly from 12 to 16, do we ordinarily find the girls in our families possessed of an alimentary, glandular and vascular system sufficiently developed and so normally active that a healthy blood, containing in quantity and quality just the pabulum needed, is carried to each tissue? During these years of puberty which we are considering, the teeth begin to break down, in not a few instances, and as a result, digestion is impaired.

But why do the teeth so often decay in girls at puberty and during early married life? Why is it, for example, that the German, Swede and Norwegian girls who come to this country with model teeth and the picture of health, unless we except the light complexion of the Scandinavians as hardly compatible with the most robust health—why is it that these girls live in our families but a few years before their teeth begin to decay, and they lose the fresh bloom of health which they brought from their native land? And then, if they marry and bear two or three children, they are almost a wreck; their teeth are so decayed as to be of no use for mastication. They are poor and feeble, pale and anæmic, old and haggard, but the shadow of their former selves; in the best possible condition to receive and develop almost any form of disease, tuberculosis and the like. Why this change? It is not simply the change in their habits of life and the climate, though these have doubtless had their influence: but I believe a far more important cause will be found in the quality of their food. The quantity of the food they consume is probably greater than what they would get in the old country; but is the quality as appropriate for the nourishment and development of *all* the tissues? I believe not. For while the carbonaceous and albumenous foods are eaten in greater quantity, the *inorganic* is deficient, and here is an important source of weakness and decay, as I believe. The inorganic portion of the wheat is largely re moved in the process of grinding, leaving only the starch and some of the gluten in the white flour which is eaten. While the brown bread from the coarser flour and from rye, which was formerly used largely in this country, and is still in most foreign countries, contained far more of the inorganic substances necessary for the development and nutrition of the bones, the teeth, and other important tissues, and as the result, the teeth erupted earlier, were better formed, and did not decay as readily, the bones were better developed, and all the tissues firmer.

During pregnancy, if the inorganic foods are not furnished the mother in sufficient quantity, the fœtus will even take it from the bones, and especially the teeth, of the mother, for all the tissues, the softest as well as the hardest, must have inorganic food, and while these resources of the mother are freely drawn upon, the deficiency may be so great that both the mother and child suffer for the want of them. But it is the rule that the mother will suffer first from the deficiency, her stomach will be disturbed, indigestion, heart-burn, and the like, and her teeth will decay and break away before the tissues of the fœtus will seem to suffer. But these symptoms may usually be readily relieved by giving the mother a sufficient quantity of the lime and magnesia salts—in other words, furnishing her with a sufficient quantity of inorganic food.

And the developing tissues of infancy and childhood need more food of all kinds, the inorganic included, than the simply working tissues of adult life. So during puberty the girls need more of this food for the proper development of the reproductive organs, a whole system of organs developing within a few years. To insure, then, a normal development of the sexual organs at puberty, there must be a suitable supply of *all* kinds of food, and not the least, the inorganic.

Among our American girls there is another important cause of imperfect development, and consequently abnormal functional activity of the generative organs, in the *style of dress* now so nearly universal, consisting essentially of the tightly fitting *corset*, with equally tight outer clothing—for even if the corset may seem to be loose when first applied, and they are never acknowledged to be tight by the wearer—yet it must and does prevent the expansion and development of the lungs and muscles of the back and abdomen. It displaces the liver and stomach upward, and presses the abdominal viscera downward upon the pelvis, compressing these organs during the important period of their development, and laying the foundation for the congestions, the pelvic inflammations, the flexions, the displacements, and perhaps even the ovarian and fibroid tumors of later years. Among girls of foreign birth the corset is not generally put on until the changes of puberty are complete, consequently its evil effects are not seen as early, usually not until several years of menstrual life, or even after marriage. But our American girls are not *dressed* without the corset, even *before* puberty, so they have its evil effects during puberty, during menstrual life, and generally during the rest of their lives.

Not only does the corset prevent the development of and displace the important organs of the thorax, abdomen and pelvis, but it, either alone, or with ingeniously contrived additions, is made to compress and prevent the development of the mammary glands, while it seems to enlarge them.

Dr. DeWolf tells us that statistics show that half of all the deaths are children under 5 years of age, and that while 40 per cent. of all the babies born in the United States die in infancy, only 18 per cent. die during the same period in Norway.

The chief reason for this frightful difference in the mortality is doubtless due to the fact that so many more mothers can nurse their children in Norway than in the United States, for I will not believe that there are many mothers in any country *who will not* nurse their children when they can. Corroborating and explaining these statistics, is the statement that 10,000,000 nursing bottles were made in this country during the last year. How many were exported and how many more imported was not stated. Neither are we told how many *tons* of infant foods were manufactured in the United States and imported during the same period. Surely, all must acknowledge that very many of our American mothers cannot nurse their children, or only for a very short period, and that this is one of the important causes producing this frightful mortality among our children. Have we explained in part the reason for this? If so, the remedy is evident.

But so long as the corset is in favor, the gynæcologist and the undertaker will thrive, and the wet nurse will be in demand.

But other systems are needed to complete the harmony necessary to the perfect development of the reproductive organs at puberty, beside the alimentary, the vascular, and the glandular, The muscular and nervous systems have most important functions to perform. Physiologists estimate that the voluntary muscles normally require from one-third to one-quarter of all the blood for their nutritive and functional activity, and *vice versa*, healthy and active muscles are required for a normal blood, which shall be rich in the pabulum needed for the developing tissues. And here our American girls usually fail as puberty approaches, their muscular exercise is restricted, they are confined within doors, and their time given to music, drawing, painting and receiving friends, instead of the more active pursuits which would continue the development of the muscular system, while it aided alimentation ; or perhaps what is worse, their time is devoted largely to study, thus calling into undue activity the nervous centres, and so developing the whole nervous system that this is ever afterward the dominant system.—*Jonr. Amer. Med. Asso.*

